# Therapeutic Effect of C-Phycocyanin Extracted from Blue Green Algae in a Rat Model of Acute Lung Injury Induced by Lipopolysaccharide

**DOI:** 10.1155/2013/916590

**Published:** 2013-03-20

**Authors:** Pak-on Leung, Hao-Hsien Lee, Yu-Chien Kung, Ming-Fan Tsai, Tz-Chong Chou

**Affiliations:** ^1^Department of Intensive Care, Chi Mei Medical Center Liou Ying Campus, Tainan, Taiwan; ^2^Department of Surgery, Chi Mei Medical Center, Liou Ying Campus, Tainan, Taiwan; ^3^Institute of Physiology, National Defense Medical Center, Taipei, Taiwan; ^4^Department of Biomedical Engineering, National Defense Medical Center, Institute of Medical Sciences, Tzu Chi University, 701 Zhongyang Road, Hualien, Taiwan

## Abstract

C-Phycocyanin (CPC), extracted from blue green algae, is a dietary nutritional supplement due to its several beneficial pharmacological effects. This study was conducted to evaluate whether CPC protects against lipopolysaccharide- (LPS-) induced acute lung injury (ALI) in rats. Rats were challenged with LPS (5 mg/kg body weight) intratracheally to induce ALI. After 3 h LPS instillation, rats were administrated with CPC (50 mg/kg body weight, i.p.) for another 3 h. Our results showed that posttreatment with CPC significantly inhibited LPS-induced elevation of protein concentration, nitrite/nitrate level, release of proinflammatory cytokines, the number of total polymorphonuclear cells in bronchoalveolar lavage fluid, and lung edema evidenced by decrease of lung wet/dry weight ratio accompanied by a remarkable improvement of lung histopathological alterations. Furthermore, CPC significantly attenuated LPS-induced myeloperoxidase activity, O_2_^−^ formation, expression of inducible nitric oxide synthase, and cyclooxygenase-2 as well as nuclear factor-kappa B (NF-**κ**B) activation in lungs. Additionally, CPC significantly downregulated proapoptotic proteins such as caspase-3 and Bax, but upregulated antiapoptotic proteins such as Bcl-2 and Bcl-XL in lungs exposed to LPS. These findings indicate that CPC could be potentially useful for treatment of LPS-related ALI by inhibiting inflammatory responses and apoptosis in lung tissues.

## 1. Introduction 

Acute lung injury (ALI), a severe complication often observed in ICU patients, has a high mortality [1]. ALI is characterized by damage to the epithelial and endothelial cells in lungs, thereby increasing pulmonary vascular permeability, pulmonary edema, and sequestration of polymorphonuclear neutrophils (PMNs), which finally impairs respiratory function. Although the true mechanisms causing ALI remain unclear, it is generally accepted that ALI is an excessive uncontrolled inflammatory response mediated by several pro-inflammatory mediators [2]. Lipopolysaccharide (LPS), a cell wall component of gram-negative bacteria, is thought to play a key role in the development of ALI through stimulation of recruitment of inflammatory cells into lungs and production of several inflammatory and chemotactic cytokines [3–5]. Although there is no ideal animal model of ALI, intratracheal (i.t.) administration of LPS into lungs is a useful and applicable experimental ALI model, which directly causes lung injury without systemic inflammation and multi-organ failure [6]. Currently, this model has been used to investigate the pathological mechanisms of endotoxin-induced ALI and evaluate the potential new therapeutic strategies.

C-Phycocyanin (CPC), a biliprotein occurred in blue green algae such as *Spirulina platensis*, is a dietary nutritional supplement due to its beneficial values, including hepatoprotective, antiplatelet, neuroprotective, antioxidant, and anti-inflammatory activities [7–10]. Our previous study has shown that in LPS-stimulated RAW 264.7 macrophages, CPC exhibits an anti-inflammatory activity by inhibiting inducible nitric oxide synthase (iNOS) expression and NO production possibly by suppressing nuclear transcription factor-*κ*B (NF-*κ*B) activation, a key transcription factor promoting pro-inflammatory gene expression [11]. These findings suggest that CPC may be a potential reagent to treat inflammatory diseases. However, there is little information on whether CPC has an ability to protect against LPS-induced ALI. The aim of this study was to investigate the effects of CPC on LPS (i.t.)-induced ALI in rats and further studied the possible mechanisms involved. Our results have indicated that CPC exhibits a therapeutic effect in LPS-induced ALI. 

## 2. Materials and Methods 

### 2.1. Animal and Reagents. 

Male Sprague-Dawley (SD) rats (8 weeks of age, 200–230 g) were used for this study. All experimental procedures were conducted in accordance with the Guiding Principle in the Care and Use of Animals and approved by the Institutional Animal Care and Use Committee of National Defense Medical Center, (Taipei, Taiwan). The CPC (with a purity of A_620_/A_280_ >3.5) provided by Far East Bio-Tec. Co., Ltd. (Taipei, Taiwan) was dissolved in normal saline. Other chemical agents used in the study were at least analytical grade.

### 2.2. LPS-Induced ALI Model and Grouping. 

The rats were divided randomly into three groups: (1) phosphate buffered saline (PBS) vehicle group (control group); (2) LPS challenge group (LPS group); (3) LPS and CPC-treated group. For intratracheal instillation, rats were anesthetized with a single intraperitoneal dose of ketamine (90 mg/kg body weight) and xylazine (7 mg/kg body weight), followed by administration of LPS (*Escherichia coli* 055:B5, 5 mg/kg body weight, i.t.) in 0.2 mL PBS through a 24-gauge catheter to induce lung injury as previously described [12]. Control rats were given 0.2 mL PBS intratracheally. The treated rats were administrated with CPC (50 mg/kg body weight) intraperitoneally (i.p.) 3 h after instillation of LPS. In our preliminary study, we have found that the pathological features of LPS-induced ALI were significantly improved if rats were treated with CPC at a dosage of 50 mg/kg. Therefore, the dosage of CPC (50 mg/kg body weight, i.p.) was chosen in the following experiments. All rats were euthanized at 6 h after LPS or PBS instillation and the samples were collected for subsequent analyses. 

### 2.3. Bronchoalveolar Lavage and Cell Counting

At the end of the experiment, the right lungs were ligated at the right main bronchus. Then, the left lungs were lavaged with 1 mL of autoclaved PBS for five times. The recovery ratio of the fluid was about 80% (4 mL). The bronchoalveolar lavage fluid (BALF) obtained from left lungs was immediately centrifuged at 300 g for 10 min at 4°C, and the supernatants were stored at −70°C until required for subsequent tests. The cell pellets were resuspended in PBS, and the total cell number was counted using a standard hemocytometer. 

### 2.4. Evans Blue Dye Leakage

Thirty minutes before the end of the experiment, Evans blue dye (50 mg/kg, Sigma-Aldrich, St. Louis, MO, USA) was given intravenously. After the lungs were perfused free of blood, the Evans blue content in lung tissue was determined using a spectrophotometer at the optical density of 620 nm.

### 2.5. Wet-to-Dry Lung Weight Ratio and Protein Content in BALF

Six hours after the intratracheal instillation of LPS or PBS, right lungs were excised and immediately weighed the wet weight. Dry weight was determined after heating the lungs in an oven at 80°C for 48 h. The wet/dry weight ratio of right lungs was measured to assess pulmonary edema. The protein content in cell-free BALF obtained from left lungs was determined by using a Bio-Rad protein assay kit (Bio-Rad Laboratories, Hemel Hempstead, UK) with bovine serum albumin (Sigma) as a standard.

### 2.6. Measurement of Cytokines and Nitrite/Nitrate (NO_*x*_)

The levels of pro-inflammatory cytokines (TNF-*α*, IL-6, and IL-1*β*) and cytokine inducible neutrophil chemoattractant-3 (CINC-3) in BALF obtained from left lungs were measured by using enzyme-linked immunosorbent assay (ELISA) kits (Genzyme Corporation, Cambridge, MA, USA), respectively. To determine the NO formation, the BALF was injected into a collection chamber containing 5% VCl_3_. In this strong reducing environment, both nitrate and nitrite were converted to NO. A constant stream of helium gas was used to carry the output into an NO analyzer (Sievers 280NOA; Sievers Instruments Inc., Boulder, CO, USA), where the NO reacts with ozone (O_3_), resulting in the emission of light. Light emission is proportional to the NO formed, and the level of NO_*x*_ (nitrite + nitrate) reflects the total amount of NO produced by nitrite + nitrate [13]. Standard amounts of sodium nitrate were used for calibration.

### 2.7. Myeloperoxidase (MPO) Activity Assay

 The right lungs were homogenized and sonicated in 50 mM potassium phosphate, (pH 6) containing 5 mM hexadecyltrimethylammonium bromide (Sigma-Aldrich, St. Louis, MO, USA), and subjected to three cycles of freezing and thawing followed by centrifugation at 14,000 g for 10 min at 4°C to obtain the supernatant. Then, 20 *μ*L tissue supernatant was incubated with 40 *μ*L assay buffer containing 0.167 mg/mL O-dianisidine dihydrochloride (Sigma-Aldrich, St. Louis, MO, USA), and 0.0005% hydrogen peroxide. The MPO activity was calculated as the change in absorbance at 460 nm over 1 min and expressed as U/g weight.

### 2.8. Measurement of O_2_^−^ Production in Lungs

The freshly harvested right lungs were cut into pieces of 5 × 5 mm and incubated with oxygenated Krebs-Hepes buffer for 5–10 min. Then, the pieces of lung tissue were transferred to 96-well microplates. These microplates containing 1.25 mM lucigenin in 200 *μ*L Krebs-Hepes buffer were placed into a microplate luminometer (MicroLumat LB96V, Berthold, Germany). Counts of chemiluminescence signal from lungs were obtained at 1 min intervals at room temperature. The counts of plates containing all components without lung tissue were regarded as background, and the blank value was subtracted from the chemiluminescence signal obtained from the lung samples. The results were expressed as count per second (CPS) per milligram dry weight (CPS/mg dry weight).

### 2.9. Immunohistochemistry Staining for Nitrotyrosine

 The right lung sections were deparaffinized, dehydrated, and immersed in 10 mM sodium citrate buffer for 5 min at 100°C. After blocking with 5% bovine serum albumin at room temperature for 20 min, the slides were incubated at 4°C with a polyclonal nitrotyrosine primary antibody (1 : 50 dilution; Santa Cruz Biotechnology, CA, USA) overnight, followed by addition of a goat anti-rabbit immunoglobulin horseradish peroxidase-conjugated secondary antibody (1 : 50; Abcam, Cambridge, UK) for 1 h at room temperature. The presence of nitrotyrosine was indicated by the brown peroxidase reaction product and photographed with light micrographs (Leica DMI6000B, Wetzlar, Germany).

### 2.10. Quantitative Real-Time PCR Assay

Total RNA was extracted from right lung tissues by using TRIzol reagent (Life Technologies, Carlsbad, CA, USA), and RNA was reverse-transcribed to cDNA using RT and amplified by PCR with *Taq* polymerase (Invitrogen, Carlsbad, CA, USA) according to the manufacturer's instructions. Relative expressions of Bax, Bcl-2, and Bcl-XL were measured with a SYBER green detection system by using ABI 7300 Real-Time PCR device (Applied Biosystems, Foster City, CA, USA). The threshold (*C*_*T*_) values for each mRNA were subtracted from that of *β*-actin mRNA, and converted from log-linear to linear term. The primer sequences used were as follows:

 Bax:
 forward 5′-CCAGGATCGAGCAGAGAGG-3′,
 reverse 5′-CGGAGGAAGTCCAGTGTCC-3′.
 Bcl-2:
 forward: 5′-TAATACGACTCACTATAGGCGGGAGATCGTGATGAAGTA-3′,
 reverse: 5′-ATTTAGGTGACACTATAGAGAAGGGCGTCAGGTGC-3′.
 Bcl-XL:
 forward 5′-GAGTTTGAGACCCGCTTCC-3′,
 reverse: 5′-GTCCTCACTGATGCCCAGTT-3′.
 
*β*-actin:
 forward 5′-GACCCAGATCATGTTTGAGACCTTC-3′,
 reverse 5′-GGTGACCGTAACACTACCTGAG-3′.

### 2.11. Western Blot Analysis

 Lung tissues were homogenized in RIPA lysis buffer (50 mM Tris-HCl, pH 7.4, 150 mM NaCl, 0.25% deoxycholic acid, 1% NP-40, and 1 mM EDTA) containing EDTA-free protease inhibitor cocktail (Thermo scientific, USA). The proteins were extracted at 4°C for 30 min and centrifuged twice at 10,000 g for 10 min at 4°C, and protein concentrations were determined by using Bio-Rad protein assay kit. The samples (100 *μ*g protein/lane) were loaded and separated on 10% sodium dodecyl sulfate (SDS) polyacrylamide (PAGE). The separated proteins were transferred to polyvinylidene fluoride membranes (Immobilon-P; Millipore, Bedford, MA, USA) using a Wet transfer tank (Bio-Rad, Hercules, CA, USA) and blocked with 5% skim milk in TBST (20 mM Tris-base, pH 7.5, 500 mM NaCl, and 0.1% Tween 20, v/v) for 1 h. After blocking, the membranes were incubated overnight at 4°C with specific primary antibodies against with cyclooxygenase-2 (COX-2, 1 : 500, BD Bioscience Pharmingen, San Diego, CA, USA), iNOS (1 : 500, Biotechnology, Sanata Cruz, CA, USA), caspase-3 (1 : 1000, Cell Signaling Technology, Danvers, MA, USA), or *β*-actin (1 : 5000) in 5% skim milk. After additional washes, the membranes were incubated with a goat anti-rabbit immunoglobulin horseradish peroxidase-coupled secondary antibody (Abcam, Cambridge, UK) at a dilution of 10,000 in 5% skim milk for 1 h. The immuno-reactive bands were visible after development with a chemiluminescence (ECL) reagent (Amersham International Plc., Buckinghamshire, UK) and were quantified by densitometry and normalized with respective *β*-actin.

### 2.12. Assay of NF-*κ*B Translocation

The lung tissue slices mounted on the coverslips were incubated with rabbit monoclonal phospho-NF-*κ*B p65 (Ser536) (Cell Signaling Technology, Danvers, MA, USA) overnight at 4°C diluted 1 : 100 in 1% BSA in TBS. After washing, coverslips were incubated with a fluorescein-isothiocyanate- (FITC-) conjugated secondary antibody (Santa Cruz Biotechnology, Santa Cruz, CA, USA) diluted 1 : 100 with 1% BSA in TBS. All coverslips were also stained with Hoechst 33258 dye in the nuclei for 10 min. After extensive washings with TBS, the coverslips were mounted onto the glass slides and photographed with a fluorescence microscope (Leica DMI6000B, Wetzlar, Germany). 

### 2.13. Lung Histological Analysis

The right lung tissues were removed and fixed with 4% (w/v) paraformaldehyde, dehydrated in graded ethanol, embedded in paraffin and tissue sections (4–6 *μ*m thick) were stained with hematoxylin and eosin (H&E) and photographed by a light microscopy. To determine the score of the lung injury, the histological images were evaluated by an investigator who was initially blinded to these research groups. The lung injury was scored according to the following principle: (1) alveolar congestion, (2) hemorrhage, (3) infiltration or aggregation of neutrophils in the airspace or vessel wall, and (4) thickness of the alveolar wall/hyaline membrane formation. Each item was graded according to a four-point scale from 0 to 3 as follows: 0 = no damage, l = mild damage, 2 = moderate damage, and 3 = severe damage [14].

### 2.14. Statistical Analysis

 The experimental data were expressed as the mean ± SEM. One-way ANOVA with post hoc Bonferroni test was used for statistical analysis. Results were considered significant difference at a value of *P* < 0.05.

## 3. Results

### 3.1. Effect of CPC on Lung Edema, MPO Activity, and Cell Accumulation in LPS-Induced ALI Rats

**Figure 1:**
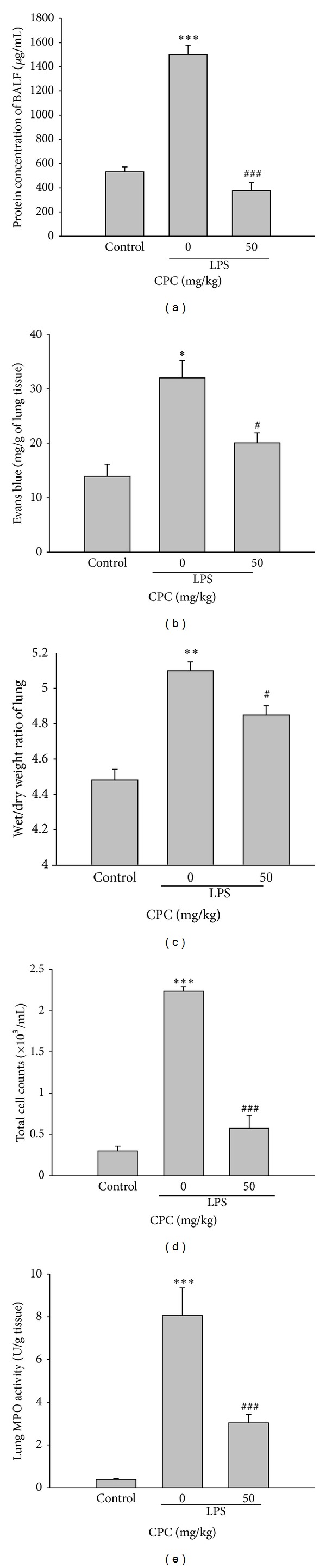
Effects of CPC on LPS-induced lung edema, lung MPO activity, and total cells in BALF. Rats were administrated with CPC (50 mg/kg, i.p.) 3 h after LPS was instilled intratracheally. The protein concentration and total cell numbers in the BALF, Evans blue level, lung wet/dry weigh ratio, and lung MPO activity were determined 6 h after LPS challenge. Data are presented as the mean ± SEM (*n* = 5). *P < 0.05, **P < 0.01, and ***P < 0.001 compared with control group; ^#^ *P* < 0.05, ^###^ *P* < 0.001 compared with LPS group.

Intratracheal instillation of LPS resulted in a significant increase in protein level and the number of total cells in BALF, Evans blue level, and lung edema evidenced by elevation of lung wet/dry weight ratio as compared to that of control rats. The elevation caused by LPS was markedly inhibited by posttreatment with CPC (50 mg/kg, i.p.) (Figure 1). The increased neutrophil infiltration is known to play an important role in the development of LPS-induced ALI, and the MPO activity was considered as a marker of influx of neutrophils. Our data also showed that elevation of lung MPO activity stimulated by LPS instillation was significantly inhibited by posttreatment of CPC (Figure 1). 

### 3.2. Effect of CPC on the Concentrations of Proinflammatory Cytokines and CINC-3 in BALF

**Figure 2:**
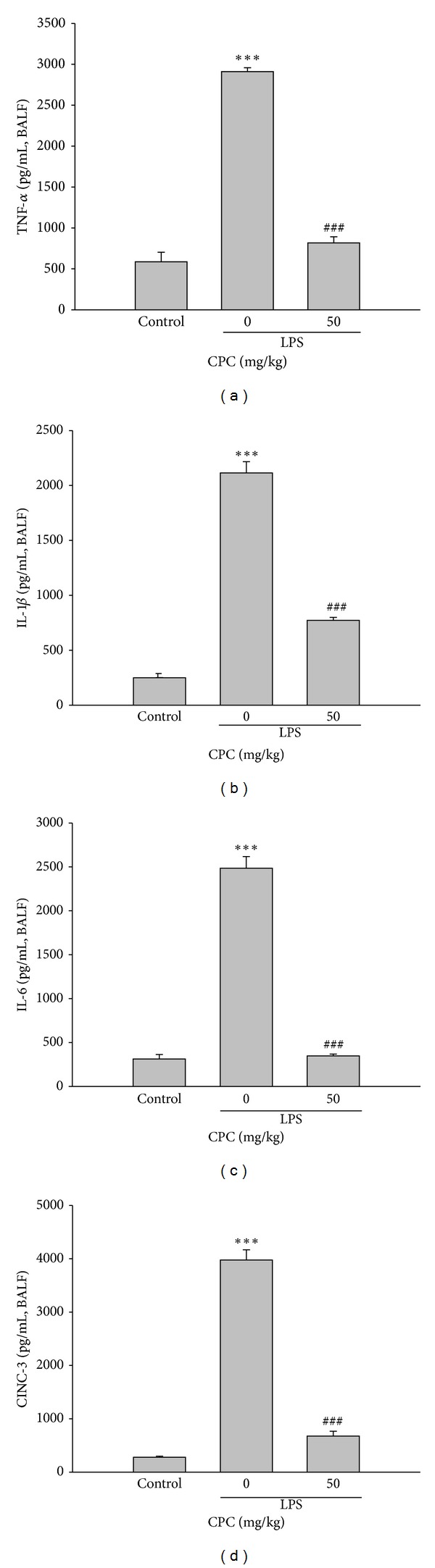
Effects of CPC on LPS-induced secretion of pro-inflammatory cytokines in BALF. BALF was prepared from rats 6 h after LPS instillation, and the levels of TNF-*α*, IL-1*β*, IL-6, and CINC-3 in the BALF were measured by ELISA kits, respectively. Data are presented as the mean ± SEM (*n* = 5). ***P < 0.001 compared with control group; ^###^ *P* < 0.001 compared with LPS group.

The levels of pro-inflammatory cytokines including TNF-*α*, IL-1*β*, IL-6, and CINC-3, a chemotactic cytokine, in BALF were markedly increased in rats received LPS instillation compared to control group. The augmentation of these inflammatory mediators induced by LPS was all significantly attenuated by posttreatment of CPC as compared to LPS-instilled alone group (Figure 2).

### 3.3. Effect of CPC on NO_*x*_, O_2_^−^ Production and Nitrotyrosine Expression in Lungs

**Figure 3:**
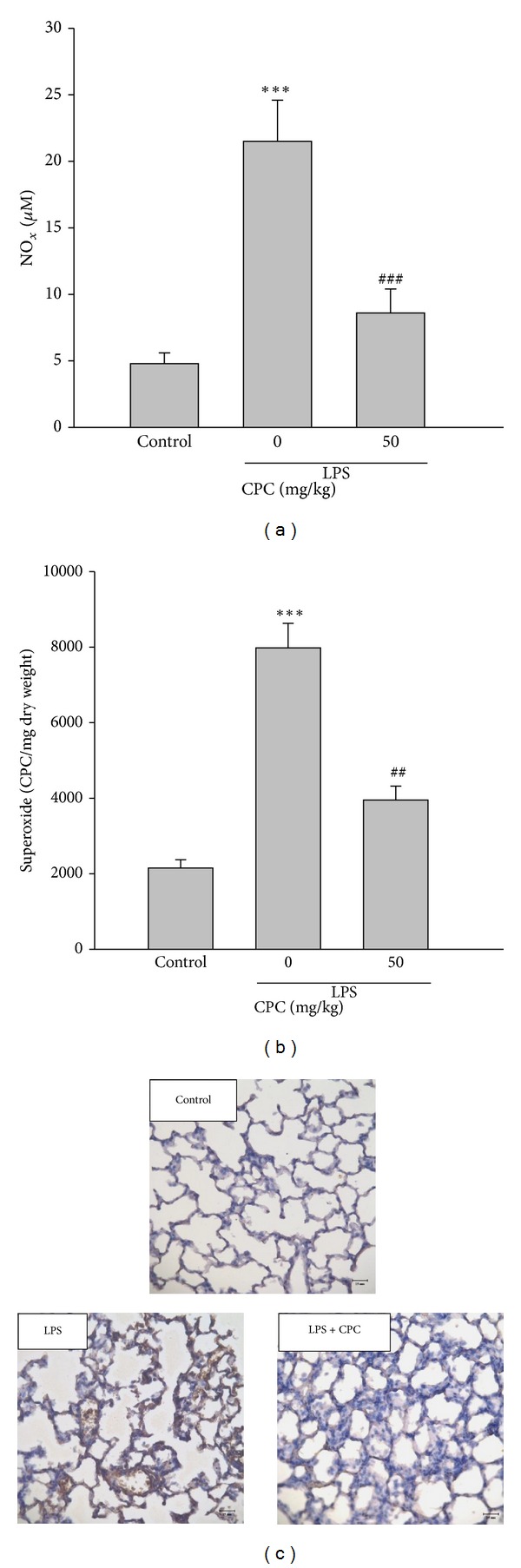
Effects of CPC on superoxide production and nitrotyrosine expression in lungs and NO_x_ formation in BALF in LPS-induced ALI rats. Rats were administrated with CPC (50 mg/kg, i.p.) 3 h after LPS was instilled intratracheally. BALF was prepared from rats 6 h after LPS challenge, and the level of NO_*x*_ was determined (a). Lung homogenates were used to measure superoxide production (b) and nitrotyrosine expression (c). Data are presented as the mean ± SEM (*n* = 5). ***P < 0.001 compared with control group; ^##^ *P* < 0.01, ^###^ *P* < 0.001 compared with LPS group.

The levels of NO_*x*_ in BALF and O_2_^−^ production (Figures 3(a) and 3(b)) and nitrotyrosine expression (Figure 3(c)) were significantly increased after LPS instillation, and these levels were significantly reduced by posttreatment with CPC. 

### 3.4. Effect of CPC on NF-*κ*B Activation and iNOS and COX-2 Expressions

**Figure 4:**
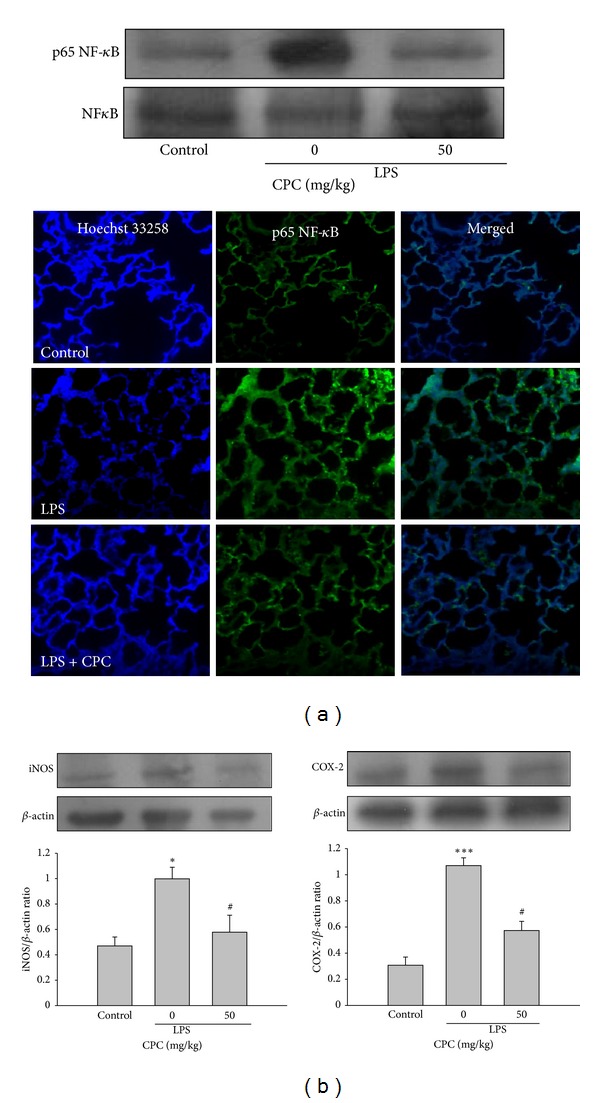
Effects of CPC on LPS-induced NF-*κ*B activation, iNOS and COX-2 expressions in lungs. The expression of nuclear phosphorylation-p65NF-*κ*B (a), iNOS and COX-2 (b) in lungs was determined by Western blot 6 h after LPS challenge. For NF-*κ*B nuclear translocation assay, the nuclei of these cells visualized by Hoechst 33285 staining (blue) and the p65NF-*κ*B expression (green) were detected in lungs. The merged figures indicated the translocation of p65NF-*κ*B to nuclei. The figures are representative of four similar experiments. Data are presented as the mean ± SEM (*n* = 4). *P < 0.05, ***P < 0.001 compared with control group; ^#^ *P* < 0.05 compared with LPS group.

Activation of NF-*κ*B is known critical for inducing several inflammatory gene expression such as iNOS and COX-2 in the response to LPS [15]. Our results showed that posttreatment with CPC greatly inhibited LPS-induced NF-*κ*B activation confirmed by the attenuation of nuclear translocation of NF-*κ*B and phosphorylation-p65NF-*κ*B expression in nuclei as compared to LPS group (Figure 4(a)). As expected, the LPS-induced protein expressions of iNOS and COX-2 in lungs were significantly inhibited by CPC (Figure 4(b)). These findings suggest that the inhibitory effects of CPC on iNOS, COX-2 expressions and pro-inflammatory mediator formation in LPS-induced ALI model may be associated with suppression of NF-*κ*B activation.

### 3.5. Effect of CPC on Apoptosis-Related Gene Expression

**Figure 5:**
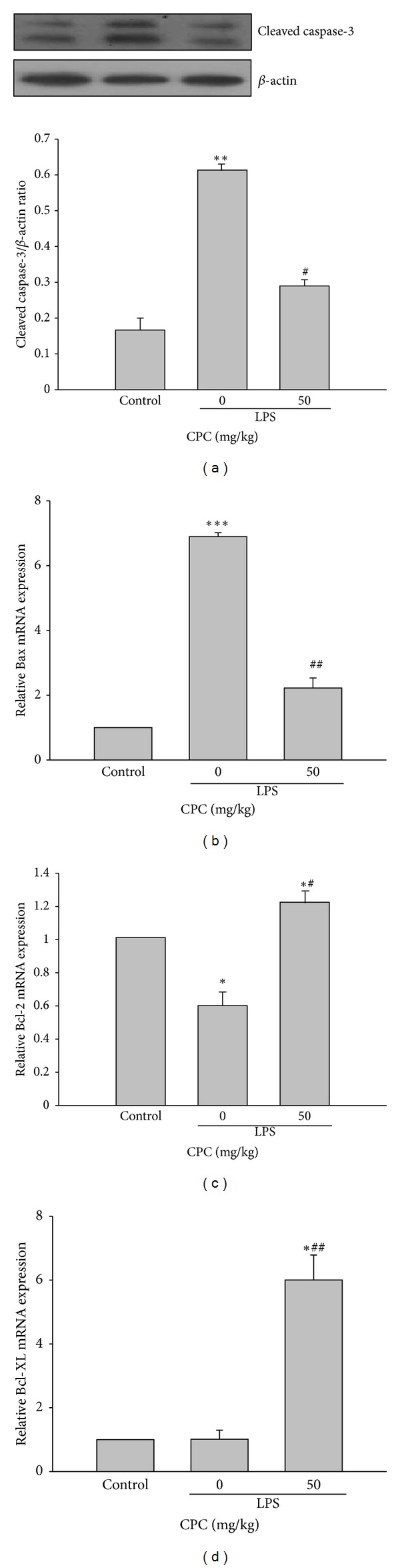
Effects of CPC on apoptosis-related protein expression in lungs from rats challenged by LPS. The cleaved caspase-3 protein expression, Bax, Bcl-2, and Bcl-XL mRNA expression levels in lungs were determined 6 h after LPS instillation. The relative expression of mRNA was expressed as folds of the control group. The expression levels from control group were assigned as 1. Data are presented as the mean ± SEM (*n* = 4). *P < 0.05, **P < 0.01, ***P < 0.001 compared with control group; ^#^ *P* < 0.05, ^##^ *P* < 0.01 compared with LPS group.

The degree of cleaved caspase-3 protein expression, a key mediator for cell apoptosis, in lungs was markedly higher in LPS-instilled rats than in control rats, and the increase was significantly inhibited by CPC (Figure 5). To further examine the mechanisms underlying the anti-apoptotic activity of CPC, the mRNA expression of pro-apoptotic (Bax) and anti-apoptotic (Bcl-2, Bcl-XL) proteins was measured. As shown in Figure 5, CPC markedly inhibited Bax mRNA expression and up-regulated Bcl-2 and Bcl-XL mRNA expressions as compared to LPS-instilled alone group. Therefore, the inhibitory effect of CPC on apoptosis may be, at least in part, attributed to the reduction of pro-apoptotic/anti-apoptotic protein ratio. Additionally, we observed that there are no toxic signs found in organs including lung in rats treated with CPC (50 mg/kg, i.p.) alone compared to the control group, suggesting that CPC is safe at the dose used. Moreover, the parameters measured in this study were all not affected by CPC treatment in the absence of LPS.

### 3.6. Effects of CPC on Lung Histopathological Changes

**Figure 6:**
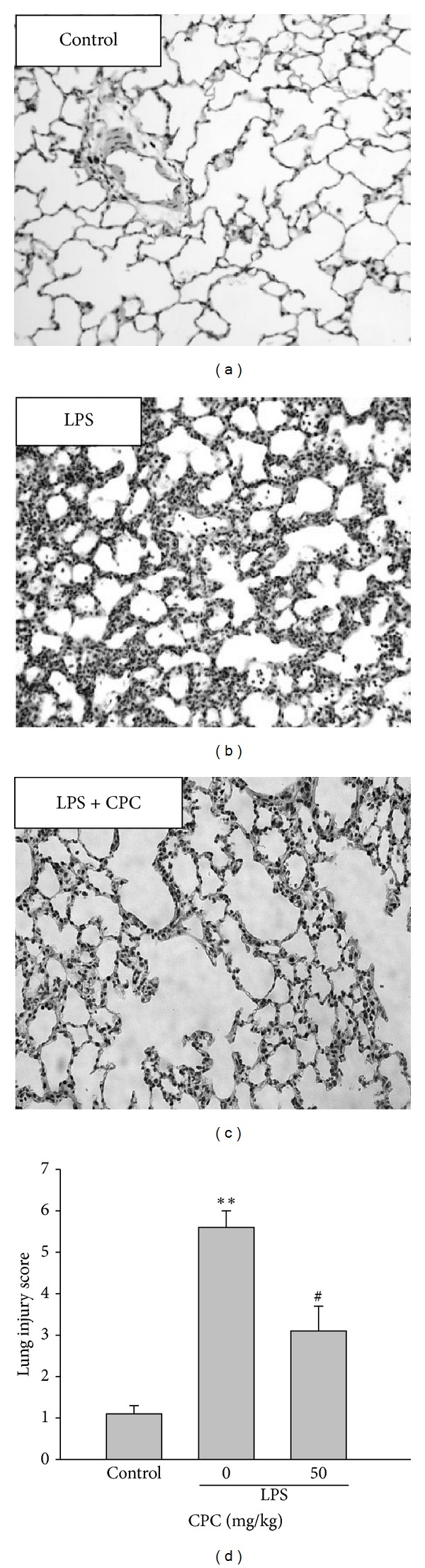
Effect of CPC on LPS-induced lung histopathological changes. Six hours after LPS instillation, the lung tissues were fixed and stained with H&E (200x magnification). The lung injury score was used to evaluate the histological alterations. Data are presented as the mean ± SEM (*n* = 4). **P < 0.01 compared with control group; ^#^ *P* < 0.05 compared with LPS group.

The lungs exposed to LPS instillation caused several histopathologicial alterations characterized by lung edema, alveolar wall thickening, and neutrophil infiltration into the lung interstitium and alveolar space. Similarly, posttreatment with CPC greatly improved these abnormal features of ALI confirmed by the lower injury score of lungs (Figure 6), indicating that CPC is a potential therapeutic drug for LPS-induced ALI.

## 4. Discussion

In this ALI model, instillation of LPS resulted in an inflammatory cascade, and in turn increasing the endothelial and epithelial permeability as well as pulmonary edema, which meets the characteristics of ALI. We demonstrated, for the first time, that posttreatment with CPC significantly reduces the level of Evans blue, an index of tissue permeability, and protein concentration in BALF and improves pulmonary histological alterations. These results support that CPC protects against LPS-induced ALI. Next, the precise mechanisms accounting for the protective effect of CPC were investigated. 

Accumulation of inflammatory cells including neutrophils in BALF has been implicated in the poor prognosis in septic ALI, and blocking recruitment of neutrophils mitigates the pathological symptoms of ALI [16]. Consistent with histological analysis of the lungs, rats challenged by LPS markedly increased the numbers of total cells in BALF and pulmonary MPO activity, a marker of neutrophil influx. Furthermore, it is known that LPS-induced acute pulmonary inflammation is characterized by release of various pro-inflammatory cytokines including TNF-*α*, IL-1*β*, and IL-6 mainly driven by neutrophils and other inflammatory cells, which subsequently amplify the inflammatory cascade and recruit neutrophils into the lungs leading to lung injury [17]. Clinical study has confirmed that in patients with ALI, the concentrations of pro-inflammatory cytokines in BALF were elevated, and this is closely related to their severity and poor outcome [18, 19]. Posttreatment with CPC could significantly attenuate lung tissue neutrophilia and levels of TNF-*α*, IL-1*β*, IL-6, and CINC-3, a chemokine, in BALF in rats challenged by LPS instillation. Therefore, inhibition of inflammatory cell sequestration and infiltration into the lungs as well as production of pro-inflammatory cytokines all contribute to the beneficial effect of CPC in LPS-induced ALI. 

NO, synthesized by NOS, is an important regulatory mediator in modulating several physiological functions. However, under stimulation of LPS and/or inflammatory mediators, the iNOS-derived NO overproduction by alveolar inflammatory cells results in the dysfunction of alveolar-capillary barrier and pulmonary edema in LPS-induced ALI [20, 21]. Clinical study has reported that the pulmonary iNOS expression and concentration of NO_x_ in BALF from patients with acute respiratory distress syndrome (ARDS) are significantly higher than those in normal subjects [22]. As expected, LPS-induced iNOS expression and NO formation were inhibited by CPC. Moreover, several studies have revealed that upregulation of COX-2 in response to LPS is thought to play an important role in the pathogenesis of inflammatory diseases such as ALI [23, 24]. Similarly, LPS-induced COX-2 expression in lungs was significantly inhibited by posttreatment with CPC. Collectively, these findings indicate that the mechanisms by which CPC mitigates LPS-induced lung injury may be related to effective abrogation of iNOS and COX-2 induction. 

It has been demonstrated that overproduction of reactive oxygen species (ROS) is crucial in the development of lung injury in response to LPS possibly through decrease of superoxide dismutase (SOD) activity, an antioxidant enzyme scavenging superoxide [25, 26]. During the inflammatory processes of ALI induced by LPS, neutrophils located in lungs and airways have been regarded as a major source to produce superoxide by undergoing a respiratory burst. What is more worse is that the overproduction of NO and O_2_^−^ can rapidly generate peroxynitrite (ONOO^−^), a highly cytotoxic product, and subsequently causes severe damage to cells and tissues. It was found that the superoxide formation and nitrotyrosine expression, a marker of peroxynitrite, in lungs exposed to LPS were attenuated by CPC administration. Therefore, it is likely that the antioxidative effect of CPC may be mediated by decreased neutrophil infiltration as a result of the suppression of pro-inflammatory cytokine production. However, we cannot exclude the possibility that the inhibition of ROS formation by CPC may be due to its direct activation of the antioxidant enzymes including SOD and glutathione peroxidase (GSH-Px) [27]. 

There are two types of cells to form the alveolar/capillary barriers, the microvascular endothelium and the alveolar epithelium. The importance of endothelial injury and increased vascular permeability in the formation of pulmonary edema is well established. To date, the alveolar and distal airway epithelium have been believed to play a critical role in regulation of alveolar fluid clearance through the actions of transepithelial Na^+^ and Cl^−^ transports [28]. The disruption of the alveolar-capillary membranes leads to alveolar flooding with serum proteins and edema fluid. A lot of evidence supports that apoptosis of endothelial and epithelial cells accompanied by increased pulmonary vascular permeability caused by a variety of inflammatory stimuli is a crucial factor resulting in pulmonary edema in ALI [4]. Administration of CPC significantly inhibited LPS-induced caspase-3 and Bax expression but up-regulated Bcl-2 and Bcl-XL proteins, suggesting that reduction of pro-apoptotic/anti-apoptotic protein ratio contributes to its anti-apoptotic activity and subsequently maintains the alveolar-capillary barrier intact. Collectively, the protective effect of CPC against LPS-induced ALI may be associated with inhibition of LPS-dependent inflammatory responses and apoptosis. 

Under inflammatory condition, NF-*κ*B is activated by phosphorylation and enters into the nucleus leading to up-regulation of downstream target genes such as iNOS, COX-2, and pro-inflammatory cytokines as well as ROS formation. Moreover, ROS also function as second messenger regulating several downstream signaling molecules, including NF-*κ*B, to amplify the inflammatory responses [29]. Importantly, activated NF-*κ*B also causes alveolar epithelial cell apoptosis by regulating gene expression related to cell death [30]. It has been confirmed that NF-*κ*B activation plays a crucial role in the pathogenesis of ALI [31]. Thus, we further examined whether CPC affects the NF-*κ*B activation. As expected, posttreatment with CPC significantly inhibited LPS-induced NF-*κ*B activation in lungs evidenced by attenuation of nuclear expression of phosphorylation-p65NF-*κ*B and nuclear translocation of NF-*κ*B. Although, the true target of CPC is unclear, we propose that suppression of inflammatory cell infiltration, ROS generation, pulmonary apoptosis, and NF-*κ*B-mediated inflammatory responses may account for the protective effect of CPC. However, the possible molecular mechanisms involved need further investigation. In conclusion, this is the first study to demonstrate that CPC possesses a therapeutic effect in LPS-induced ALI through suppressing pulmonary apoptosis and NF-*κ*B-mediated inflammatory responses. Accordingly, CPC can be considered a potential drug to improve pulmonary inflammatory diseases induced by direct injury or systemic infection.
